# Membranous S100A10 involvement in the tumor budding of colorectal cancer during oncogenesis: report of two cases with immunohistochemical analysis

**DOI:** 10.1186/s12957-020-02075-4

**Published:** 2020-11-07

**Authors:** Kazumori Arai, Hisato Ishimatsu, Tomohiro Iwasaki, Chinatsu Tsuchiya, Akihiro Sonoda, Ko Ohata

**Affiliations:** 1 Department of Pathology, Shizuoka General Hospital, 4-27-1 Kitaando, Aoi-ku, Shizuoka, 420-0881 Japan; 2 Department of Gastroenterological Surgery, Shizuoka General Hospital, 4-27-1 Kitaando, Aoi-ku, Shizuoka, 420-0881 Japan; 3 Department of Clinical Research, Shizuoka General Hospital, 4-27-1 Kitaando, Aoi-ku, Shizuoka, 420-0881 Japan

**Keywords:** S100A10, Annexin A2, Tumor budding, Poorly differentiated cluster, Colorectal cancer, Immunohistochemistry

## Abstract

**Background:**

Tumor budding (TB) and poorly differentiated clusters (PDCs) are a sequence of histologic findings that predict worse prognosis and node metastasis in colorectal cancer (CRC). TB and PDC (TB/PDC) are caused by cancer cell detachment and are distinguished by the number of cancer cells that constitute a cell cluster. In short, PDC is regarded as the previous step of TB. TB/PDC and epithelial-mesenchymal transition (EMT) are closely linked, but its pathogenic mechanisms are still unclear. S100A10, a member of the S100 protein family, forms a heterocomplex with annexin A2 (ANX A2) and then translocates to cell membrane from the cytoplasm and plays various roles in cell dynamics, including plasminogen activation. S100A10 is the activation modulator of the heterocomplex and promotes cell invasion. S100A10 is involved in the remodeling of both actin and extracellular matrix (ECM), which is also associated with EMT.

**Case presentation:**

In two representative cases of conventional advanced CRC, we immunohistochemically examined S100A10 and ANX A2 expressions in which both TB and PDC were prominent. Both CRCs metastasized to multiple regional lymph nodes. In both cases, a membranous positivity for S100A10 was diffusely found in both tumor buds and PDCs and was observed in the tumor cells protruding toward the stroma, giving rise to TB/PDC. However, even in tumor glands with TB/PDC, the tumor cells with a smooth border around the stroma showed either cytoplasmic fine-granular expression or no positivity. The immunoreactivity for ANX A2 was almost the same as that for S100A10. In the main tumor components without TB/PDC, no distinct positivity was detected at their smooth borders.

**Conclusions:**

During oncogenesis, membranous S100A10 has the potential to be related to TB of CRC. This may be due to plasminogen activation, actin remodeling, and interaction with an altered ECM. However, further study is required to confirm this hypothesis.

## Background

One of the major causes of cancer-related death is colorectal cancer (CRC) [1]. In CRC, tumor budding (TB) and poorly differentiated clusters (PDCs) are new histologic scoring systems that provide more effective prognostic information independently of conventional tumor grading systems, including the tumor, node, and metastasis (TNM) staging system [2, 3]. TB and PDC (TB/PDC) are caused by the detachment of cancer cells from the main tumor body [2–4]. In CRC with prominent TB or PDC, lymph node metastasis and lymphovascular invasion are significantly higher [2, 3, 5, 6] and have the worst prognosis among other cancer types at the same TNM stage [2, 3]. Therefore, TB/PDC may also influence the choice of therapeutic management [3, 5]. TB/PDC is mainly observed at the invasion front of CRC [2–6] and are empirically mixed with each other as a sequence of histologic findings [3, 6]. Both are distinguished by the number of cancer cells (TB, < 5 neoplastic cells; PDC, ≥ 5 neoplastic cells, evaluated on hematoxylin and eosin (H&E)-stained slides) that constitute a cell cluster lacking a glandular structure [2, 3, 5, 6]. In other words, PDC is regarded as the previous step of TB [3, 6]. TB/PDC and epithelial-mesenchymal transition (EMT) are closely linked [5, 7–9]; however, its pathogenic mechanisms are still unclear. We believe that understanding the protein(s) involved in TB/PDC will help suppress the aggressiveness of CRC.

The S100 protein family, which is composed of 21 members, belongs to the superfamily of calcium-binding proteins [10]. This protein family shows cell-specific expression and has varied functions in cellular processes, such as proliferation, differentiation, and motility/invasion [10]. Furthermore, changes in the expressions and/or functions of the S100 proteins are key steps in cancer development or progression [10]. S100A10, a member of the S100 protein family, is also expressed in various cells, including cancer cells [11, 12]. S100A10 forms a heterocomplex with cytoplasmic annexin A2 (ANX A2), then translocates to the cell membrane from the cytoplasm, and plays various roles in cell dynamics [11, 12]. It is well known that the S100A10-ANX A2 heterocomplex functions as a plasminogen receptor and promotes cell migration/invasion [11–17]. However, several studies have indicated that the S100A10 subunit is the activation modulator of the heterocomplex and directly and specifically plays various roles, including plasminogen activation [12–18]. Furthermore, S100A10 regulates cytoskeletal actin remodeling and facilitates cell spreading [18, 19]. A recent study indicated the involvement of S100A10 in EMT [20]. Regarding its relationship with TB/PDC of CRC, S100A10, and ANX A2 are related not only to poor differentiation but also to the budding of a special type of cancer cells, namely, polyploid giant cancer cells (PGCCs) [21].

We hypothesized that S100A10 may also be involved in the TB of conventional CRC. We immunohistochemically examined the expressions of S100A10 and ANX A2 in two representative cases of conventional advanced CRC in which both TB and PDC were prominent.

Ethics committee of Shizuoka General Hospital approved the research on S100 protein (approval number: SGH IRB#2018091/1).

## Case presentation

### Case 1

#### Case history

A 70-year-old woman presented with bloody stool for 4.5 months. A sub-circumferential ulcerated tumor and severe stenosis in the sigmoid colon were also revealed by colonoscopy. She had no notable medical history, including cancer, or family history, and had no previous history of bloody stool. Enhanced computed tomography (CT) demonstrated the enhancing irregular thickening of the sigmoid colon wall and indicated enlargement of four regional lymph nodes. No metastasis to the other organs was detected. Regarding tumor markers, carcinoembryonic antigen (CEA) level was slightly higher at 5.5 ng/ml (normal range, 0–5 ng/ml), but the cancer antigen 19-9 (CA19-9) level was normal at 13.2 U/ml (normal range, 0–37 U/ml). Colonoscopic biopsy revealed an adenocarcinoma with a tubular growth pattern; hence, laparoscopic sigmoidectomy and lymph node dissection were performed. After the surgery, the patient received four cycles of XELOX (oxaliplatin combined with capecitabine) for 3 months; the CEA level returned to normal during this period. Mismatch repair (MMR) was genetically evaluated, and the tumor showed proficient MMR. KRAS mutational status was not analyzed. Subsequent chemotherapy was discontinued upon the patient’s request. Fortunately, the patient did not experience recurrence during the follow-up period of 21 months after surgery.

#### Pathology

TB or PDC scoring is performed by counting the number of tumor buds or PDCs under a ×20 objective lens in H&E-stained slides, respectively [3, 4, 6]. Since a ×20 objective lens with a field area of 1.227 mm^2^ was usually used, the counts of tumor buds and PDCs normalized to the recommended field of 0.785 mm^2^ for each scoring [3, 4]. Immunohistochemistry of S100A10, ANX A2, and pan-cytokeratin (pan-CK) was performed with serial tissue sections using Leica Bond-Max (Leica Biosystems, Melbourne, Victoria, Australia). The establishment, characterization, and staining protocol of S100A10 antibody have been previously described [22, 23]. The characterization and staining protocol of ANX A2 antibody have been described elsewhere [22, 24]. In immunostaining for pan-CK, the mouse monoclonal antibody multi-cytokeratin (cocktail of two clones, AE1/AE3, Leica Microsystems, Newcastle Upon Tyne, Tyne and Wear, UK) was used at 1:200 dilution, after a 5-min pretreatment with 0.4 mg/mL proteinase K (Dako, Glostrup, Denmark). In TB assessment, it should appear more visible using a pan-CK immunostain; unlike PDC, tumor buds are often difficult to distinguish from the surrounding inflammatory or stromal cells [5, 25].

##### Routine pathological findings

**Fig. 1:**
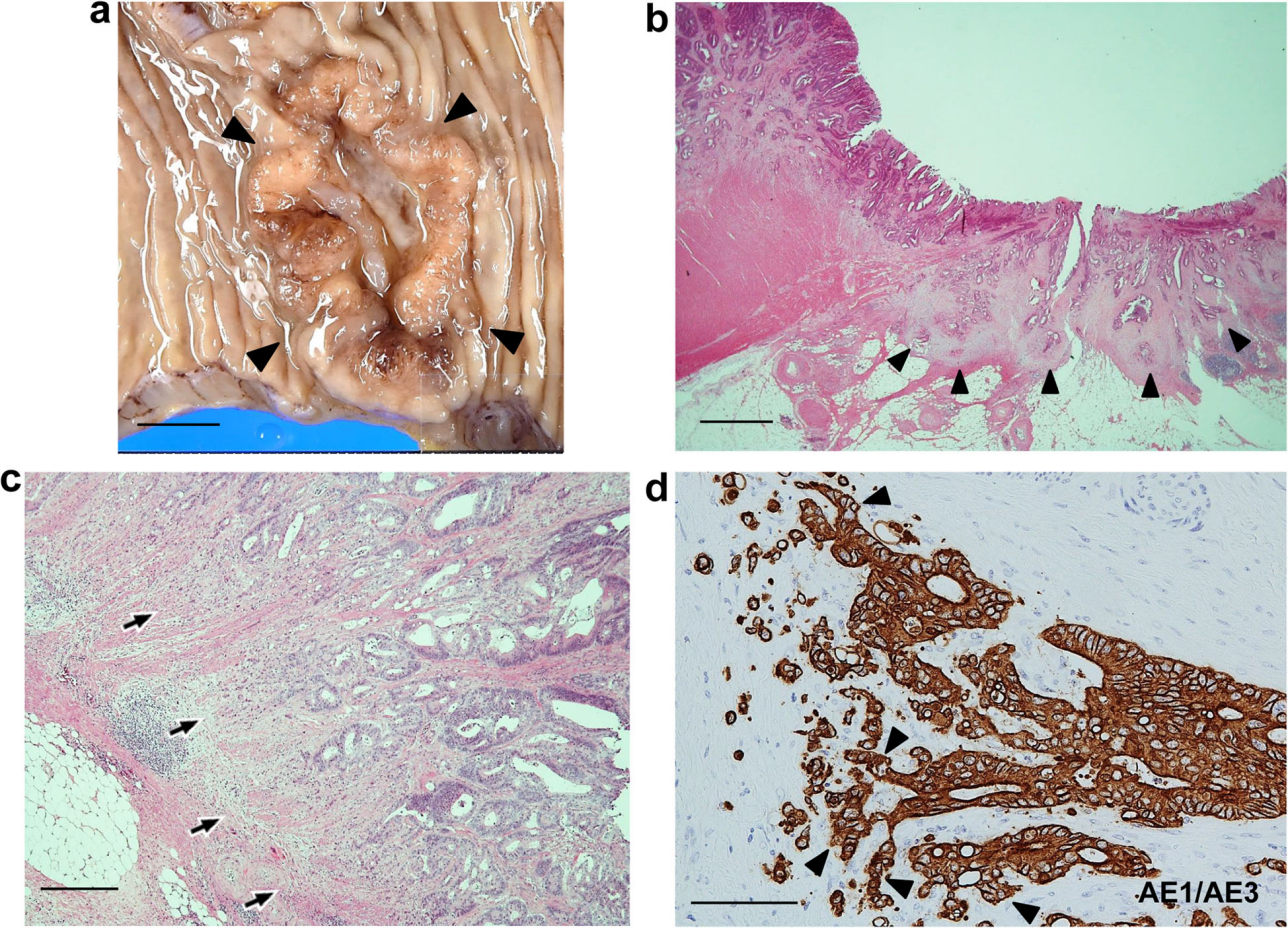
Routine pathological findings of the colon cancer in case 1. **a** Gross view shows an ulcerated tumor (surrounded by arrowheads). Scale bar: 1 cm. **b** The tumor invades the subserosa (arrowheads). Hematoxylin and eosin (H&E) stain. Scale bar: 2 mm. **c** The tumor consists mainly of an adenocarcinoma with a tubular growth pattern, but the cell aggregates with poor luminal formation are also mainly scattered in the invasive front (arrows). H&E stain. Scale bar: 400 μm. **d** Scattered cells are also strongly positive for pan-cytokeratin, and the changes in the shape of the tumor glands are more visible with immunostaining. Arrowheads indicate the tumor cell protrusion into the stroma. AE1/AE3 immunostain. Scale bar: 100 μm

Macroscopic examination showed an ulcerated tumor measuring 2.9 × 3.7 cm (Fig. 1a). Microscopically, the tumor invaded the subserosa (Fig. 1b), but no serosal exposure was noted. The primary tumor component was an adenocarcinoma with a tubular growth pattern (Fig. 1c). The aggregates of atypical cells with poor luminal formation were scattered mainly in the invasive front (Fig. 1c). In that part, the atypical cells infiltrated the stroma in isolation or in the form of small clusters of less than five cells or more. Immunohistochemically, these cells were diffusely and strongly positive for pan-CK, being regarded as tumor buds or PDCs of the tumor (Fig. 1d). The normalized maximal numbers of tumor buds and PDCs were 22 and 12 per field, respectively. Both of these numbers corresponded to score 3 [3, 4], and TB was predominant. In the tumor glands giving rise to TB/PDC, the shape appeared more irregular and protruded into the stroma in a cord-like or small nest appearance. These changes were more clearly observed on a pan-CK immunostain (Fig. 1d). In the tumor, the lymphatic invasion was noticeable, and metastases were detected in five regional lymph nodes, corresponding to pathological stage IIIB (pT3N2aM0) [26].

##### Immunohistochemical findings of S100A10 and ANX A2

**Fig. 2:**
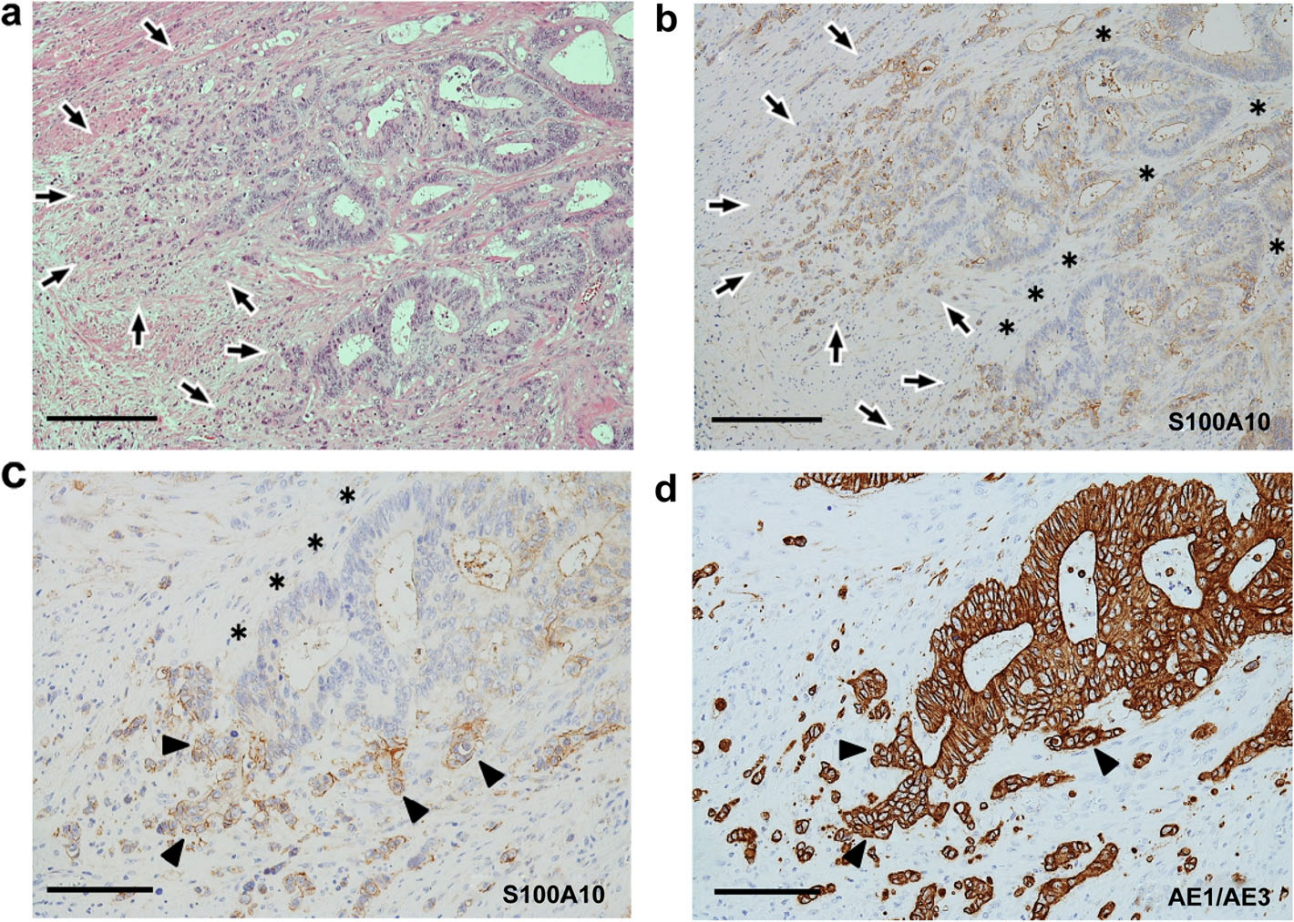
Immunohistochemistry of the level of S100A10 expression in case 1. **a** Aggregates of tumor buds and PDCs are found mainly in the left image (surrounded by arrows). Hematoxylin and eosin stain. Scale bar: 200 μm. **b** Image corresponds to (**a**). Positive reactions are noted not only in both tumor buds and PDCs (surrounded by arrows) but also in the tumor cells protruding into the stroma. By contrast, the glandular components (asterisks) with a smooth border around the stroma show no or weak cytoplasmic positivity, except for a reaction at their luminal surface. S100A10 immunostain. Scale bar: 200 μm. **c** Magnified image of (**b**). Positive reactions in the protruding tumor cells are indicated by arrowheads, and the smooth border of glandular components is indicated by asterisks. S100A10 immunostain. Scale bar: 100 μm. **d** Image corresponds to (**c**). Cord-like or small nest appearance of the protruding tumor cells (arrowheads) is more visible with pan-cytokeratin immunostain. AE1/AE3 immunostain. Scale bar: 100 μm

**Fig. 3:**
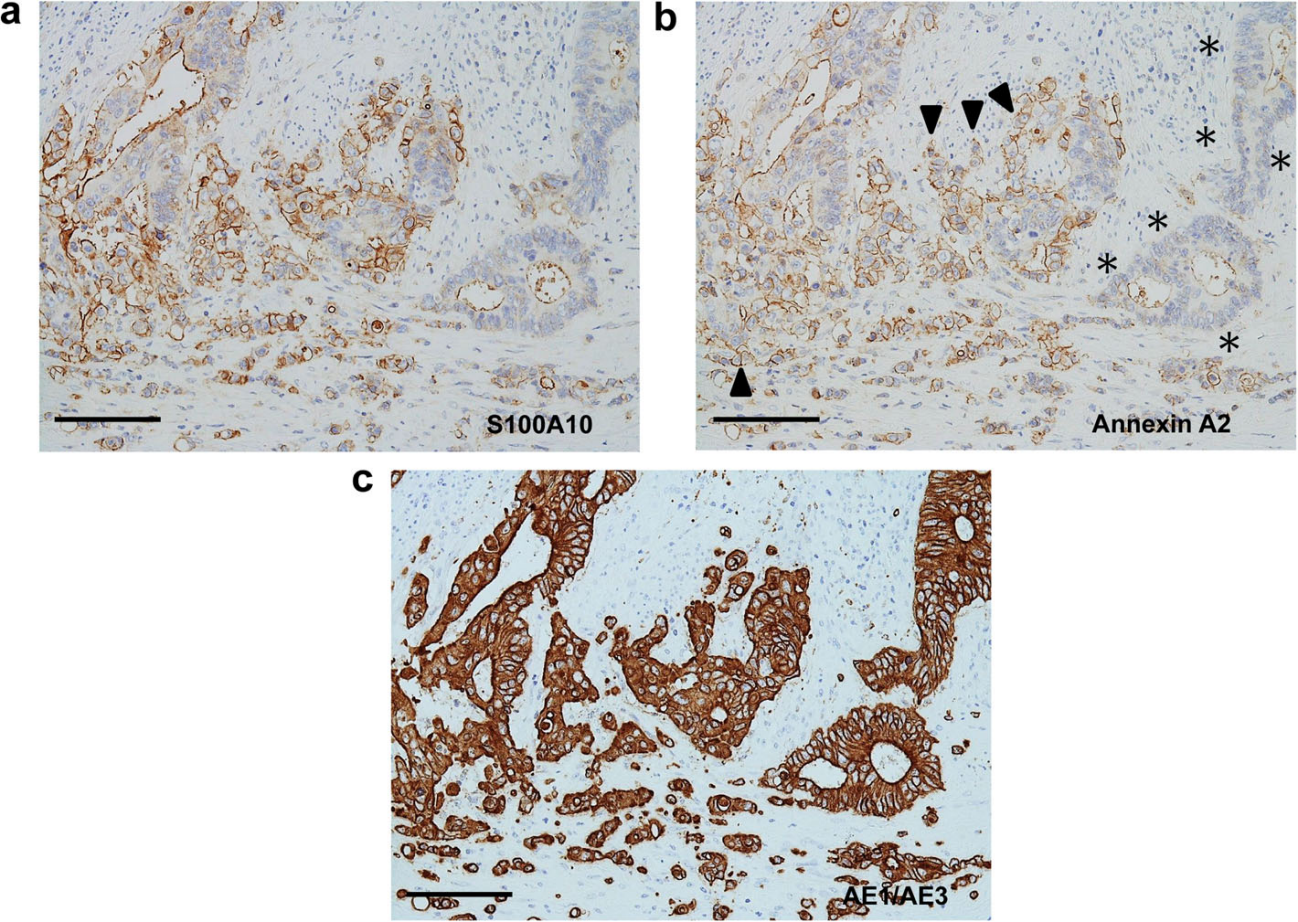
Immunohistochemical comparison between S100A10 and annexin A2 (ANX A2) in case 1. I: Tumor components with tumor buds and PDCs. **a** Immunolocalization is basically similar to those in Fig. 2b and c. S100A10 immunostain. **b** Image corresponds to (a). Immunoreactivity for ANX A2 is almost the same as that for S100A10. Arrowheads and asterisks indicate the same information presented in Fig. 2c, respectively. ANX A2 immunostain. **c** Image corresponds to both (**a**) and (**b**). The changes in the shape of tumor glands are more visible with pan-cytokeratin immunostain. AE1/AE3 immunostain. Bars: 100 μm

**Fig. 4:**
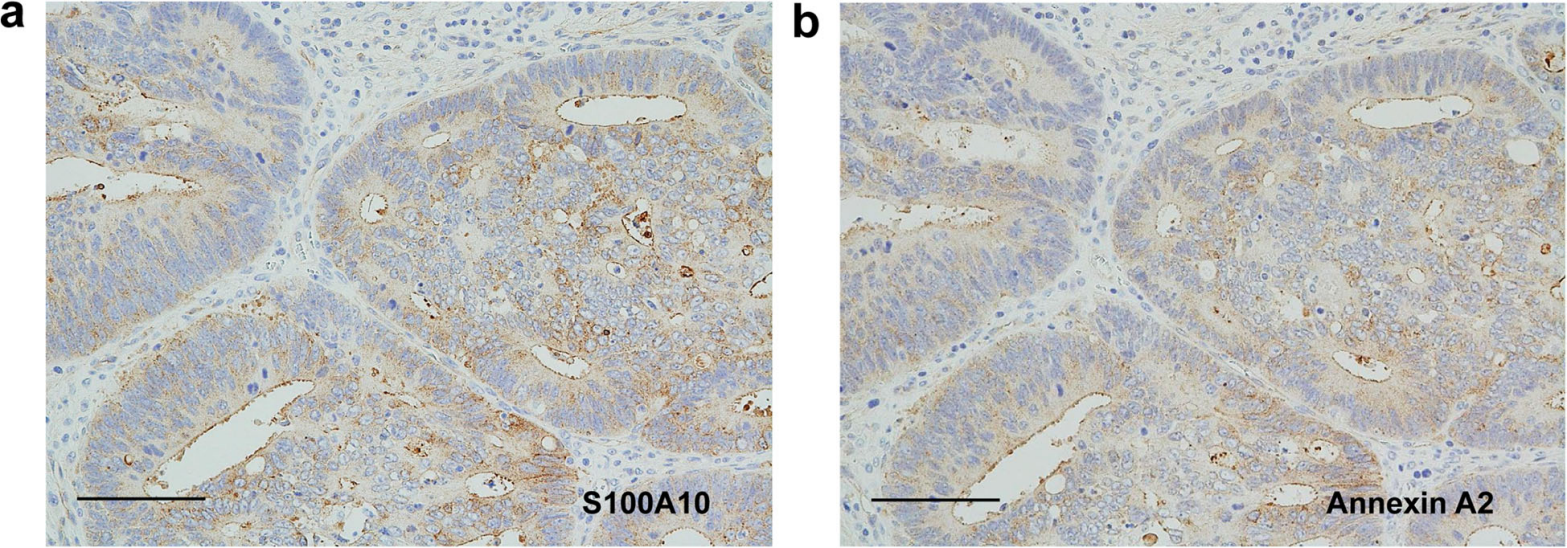
Immunohistochemical comparison between S100A10 and ANX A2 in case 1. II: Surface tumor components without tumor buds and PDCs. **a** The tumor components show a weak cytoplasmic positivity and a membranous positive reaction at their luminal surface. S100A10 immunostain. **b** Image corresponds to (**a**). Immunoreactivity for ANX A2 is almost the same as that for S100A10. ANX A2 immunostain. Scale bars: 100 μm

Membranous positivity for S100A10 was diffusely found in both tumor buds and PDCs (Fig. 2). The immunopositivity was observed in the tumor cells protruding toward the stroma (Fig. 2). However, even in tumor glands with TB or PDCs, tumor cells with a smooth border around the stroma showed either a cytoplasmic fine-granular expression or no positivity, except for a reaction at their luminal surface (Fig. 2b and c). Spindle-shaped stromal cells were negative or faintly positive for S100A10 (Figs. 2b, c, and 3a). The immunoreactivity for ANX A2 was almost the same as that for S100A10 (Fig. 3). In the main tumor components without TB/PDC, neither distinct membranous positivity for S100A10 nor for ANX A2 was seen, except for a reaction at their luminal surface (Fig. 4).

### Case 2

#### Case history

A 65-year-old man presented with bloody stool for 1 month. Patient’s colonoscopy revealed a circumferential ulcerated tumor and severe stenosis in the sigmoid colon. He had no notable medical history, including cancer, or family history, and had no previous history of bloody stool. Enhanced CT revealed an increasing irregular thickening of the sigmoid colon wall. Enlargement of the lymph nodes and metastasis to the other organs were not noted. CEA and CA19-9 levels were normal (3.9 ng/ml and 16 U/ml, respectively). Laparoscopic sigmoidectomy and lymph node dissection were performed. After the surgery, the patient received chemotherapy for 5.5 months: six cycles of XELOX and two cycles of capecitabine only. However, on magnetic resonance imaging 12 months after surgery, multiple metastases to the liver and para-aortic lymph nodes were detected. The CEA level was higher at 13 ng/ml, but the CA19-9 level remained normal. Genetically, the tumor exhibited proficient MMR. KRAS mutational status also was analyzed but was not detected. One month later, he was transferred to Shizuoka Cancer Center. The patient received chemotherapy for 16 months: 25 cycles of 5-fluorouracil combined with levofolinate and irinotecan (FOLFIRI) with panitumumab and 6 cycles of FOLFIRI alone. The metastases in the liver and lymph nodes are partially reduced, respectively, and the patient survived 30 months after surgery.

#### Pathology

##### Routine pathological findings

**Fig. 5:**
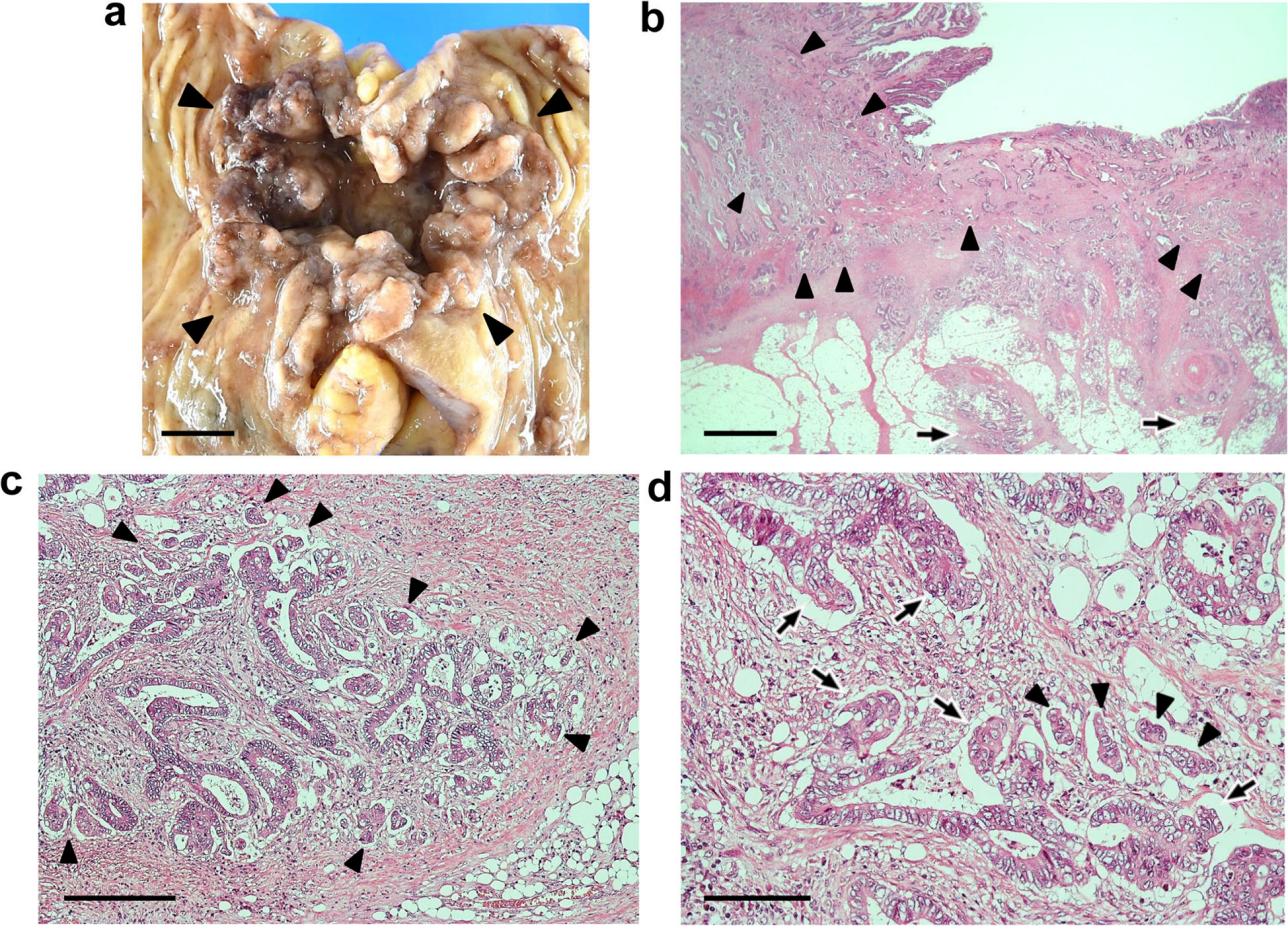
Routine pathological findings of the colon cancer in case 2. **a** Gross view shows a circumferential ulcerated tumor (surrounded by arrowheads). Scale bar: 1 cm. **b** The tumor invades the subserosa (arrows). The tumor consists mainly of an adenocarcinoma with a tubular growth pattern, but many of the cell clusters with lacunar space are distributed in the invasion front and inside the tumor (arrowheads). Hematoxylin and eosin (H&E) stain. Scale bar: 2 mm. **c** The cell clusters with lacunar space are scattered around the tumor glands, and many of them consist of more than five tumor cells, regarded as PDCs (arrowheads). H&E stain. Scale bar: 200 μm. **d** In the tumor glands giving rise to PDCs, the shape appears more irregular, protruding into the stroma in a cord-like or small nest appearance (arrows). Arrowheads indicate PDC. H&E stain. Scale bar: 100 μm

Macroscopic examination showed an ulcerated tumor measuring 5.2 × 5.5 cm (Fig. 5a). Microscopically, the tumor invaded the subserosa (Fig. 5b) and was exposed to the serosa. The tumor consisted mainly of an adenocarcinoma with a tubular growth pattern (Fig. 5b), and many PDCs with tumor buds were observed around the tumor glands (Fig. 5c). PDCs were distributed in the invasion front and inside the tumor (Fig. 5b). Many of the PDCs had lacunar spaces, showing a micropapillary carcinoma-like appearance (Fig. 5c). The normalized maximal numbers of tumor buds and PDCs were 15 and 21 per field, respectively. Both of these numbers corresponded to score 3 [3, 4], and PDC was predominant. In the tumor glands giving rise to PDCs, the shape appeared more irregular, protruding into the stroma in a cord-like or small nest appearance (Fig. 5c and d). The tumor metastasized to four regional lymph nodes, corresponding to pathological stage IIIC (T4aN2aM0) [26].

##### Immunohistochemical findings of S100A10 and ANX A2

**Fig. 6:**
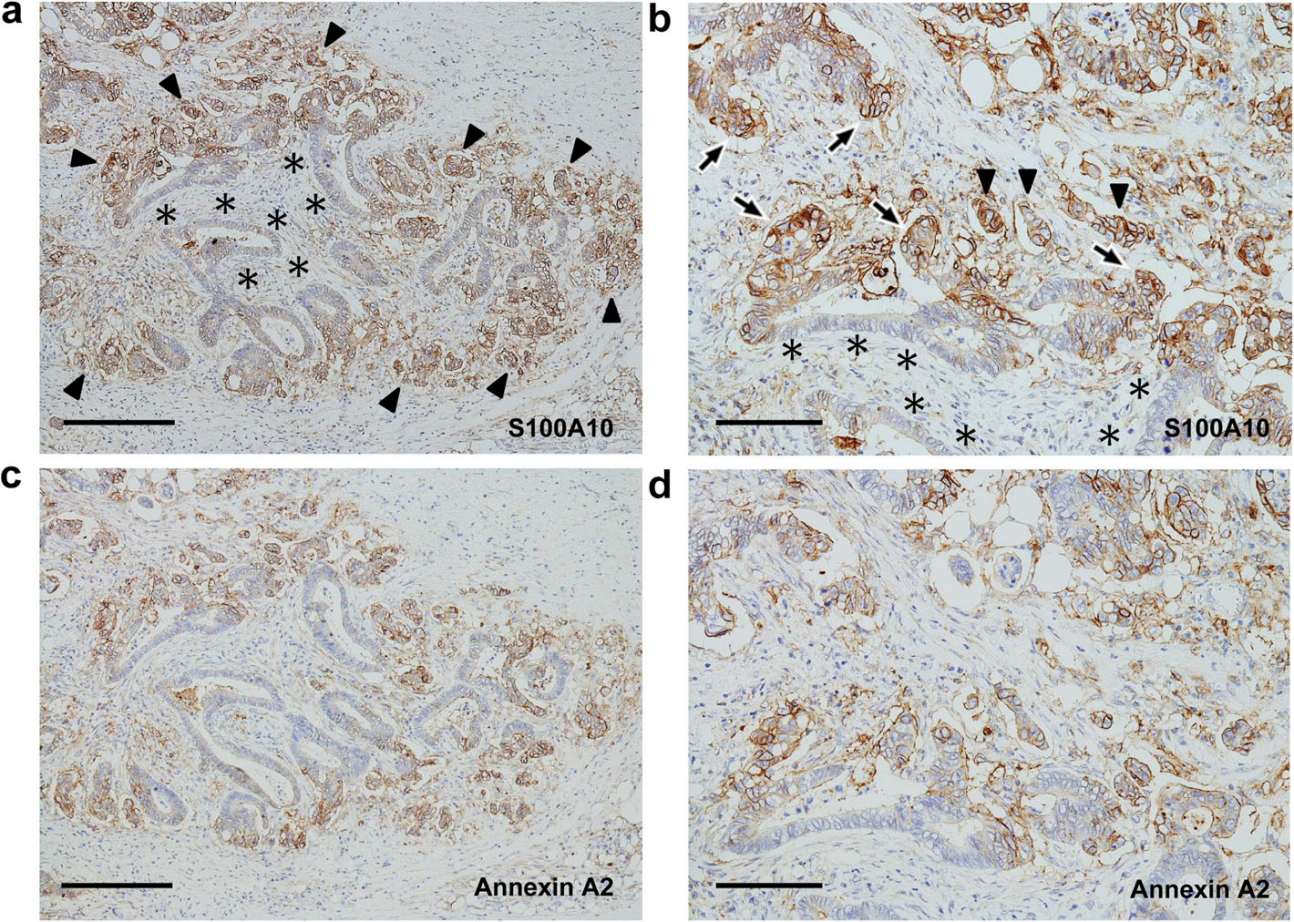
Immunohistochemical comparison between S100A10 and ANX A2 in case 2. I. **a** Positive reactions are seen not only in both PDCs (arrowheads) and tumor buds but also in the tumor cells protruding into the stroma. By contrast, the glandular components (asterisks) with a smooth border around the stroma show no or weak cytoplasmic positivity except for a reaction at the part of their luminal surface. S100A10 immunostain. Scale bar: 200 μm. **b** Magnified image of (**a**). Arrows and arrowheads indicate the protruding tumor cells and PDCs, respectively. The smooth border of glandular components is indicated by asterisks. S100A10 immunostain. Scale bar: 100 μm. **c** Image corresponds to (**a**). Immunolocalization for ANX A2 is basically similar to that for S100A10. ANX A2 immunostain. Scale bar: 200 μm. **d** Image corresponds to (**b**). ANX A2 immunostain. Scale bar: 100 μm

**Fig. 7:**
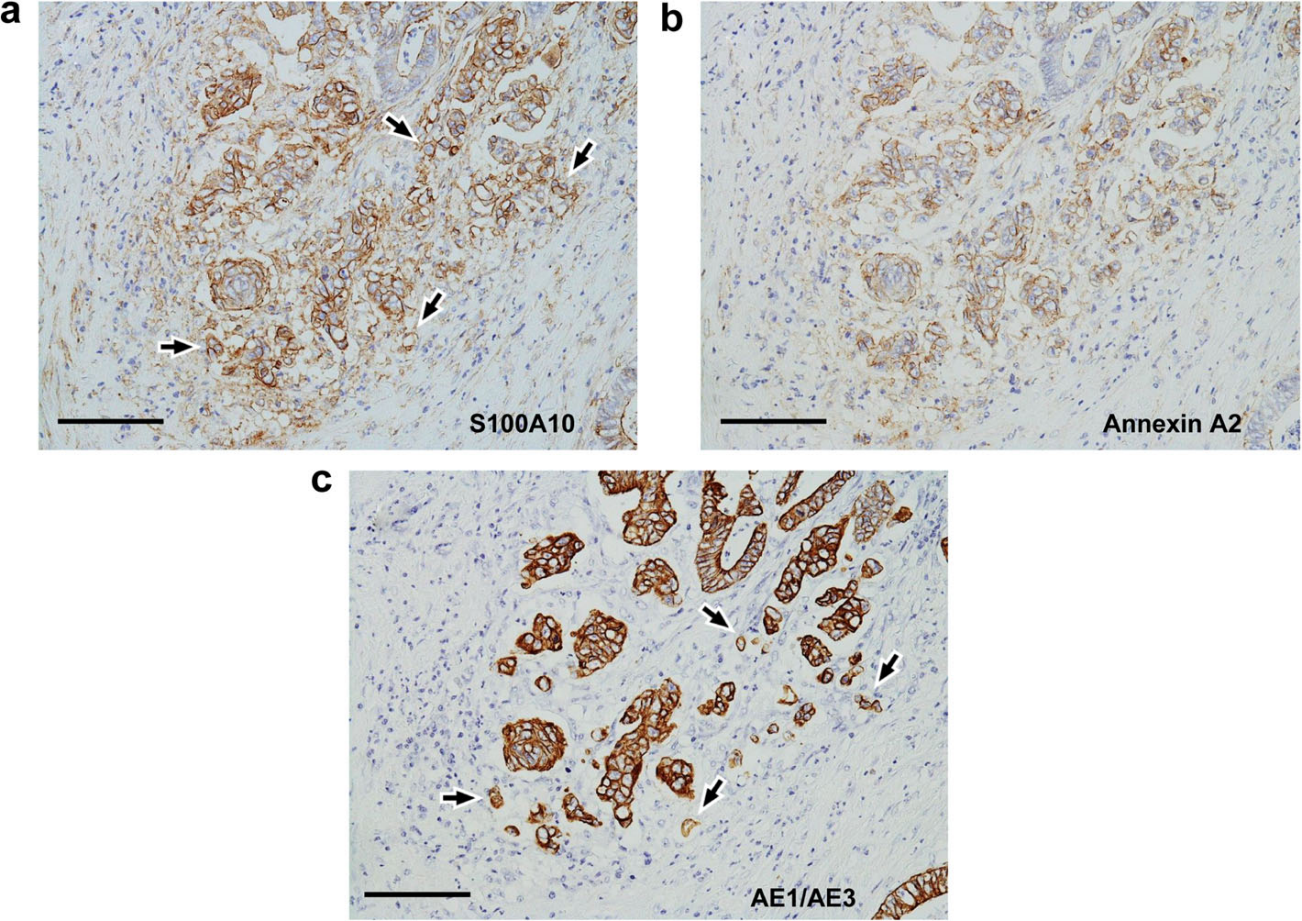
Immunohistochemical comparison between S100A10 and ANX A2 in case 2. II. **a** Tumor buds (arrows) mixed in PDCs are also positive for S100A10, as well as PDCs. S100A10 immunostain. **b** Image corresponds to (**a**). Immunoreactivity for ANX A2 is basically similar to that for S100A10. ANX A2 immunostain. **c** Image corresponds to both (**a**) and (**b**). Tumor buds (arrows) are more visible with pan-cytokeratin immunostain. AE1/AE3 immunostain. Bars: 200 μm

Membranous positivity for S100A10 was diffusely found in PDCs (Fig. 6a and b). The immunopositivity was observed in the tumor cells protruding into the stroma (Fig. 6a and b). By contrast, even in tumor glands with PDCs, tumor cells with a smooth border around the stroma showed either cytoplasmic fine-granular expression or no positivity (Fig. 6a and b). The immunoreactivity for ANX A2 was almost the same as that for S100A10 (Fig. 6c and d). Similarly, the immunopositivities for both proteins were also noted in tumor buds mixed in PDCs (Fig. 7). Furthermore, a part of the luminal surface of tumor glands showed a positivity for S100A10 and ANX A2 (Fig. 6b and d). Spindle-shaped stromal cells were faintly positive or negative for S100A10 and ANX A2 (Figs. 6 and 7). In the main tumor components without TB/PDC, neither distinct membranous positivity for S100A10 nor for ANX A2, were noted except for a reaction at their luminal surface (data not shown).

## Discussion and conclusions

In this report, the immunolocalization of S100A10 expression during the TB process in two cases of CRC was observed. Both CRCs were advanced-stage cancers with multiple lymph node metastases. The tumor in case 2 had more predominant PDCs than that in case 1; however, membranous S100A10 expression was also found in PDCs, that is, the previous step of TB, regardless of their location. In both cases, its expression was also observed in the protruding tumor cells giving rise to TB. This finding suggests that membranous S100A10 is involved in TB of CRC during oncogenesis.

PGCC is an established cancer cell line, which is induced by cobalt chloride or paclitaxel and develops in various organs, including the colon and rectum [21, 27]. A close association among PGCC, TB/PDC, EMT, and tumor differentiation has been suggested [27]. Furthermore, it was reported that S100A10 and ANX A2 are highly expressed in PGCCs with budding [21]. Our case report suggests that S100A10 and ANX A2 are also involved in TB/PDC of conventional CRC in the human body.

Considering TB/PDC, the EMT process of cancer cells can be helpful [7, 8, 28, 29]. In the EMT, the following phenomena occur in cancer cells: activation of several signal transduction pathways, such as RAS, Wnt, and TGF-β; morphological changes followed by the cytoskeleton remodeling; reduction of the contacts with cancer cells or with extracellular matrix (ECM); cancer cell detachment from the main tumor body; degradation of ECM and interaction with altered ECM; and enhancement of cell migration/invasion capacity [28, 29]. Cancer-associated fibroblasts (CAF) were also reported to contribute to these phenomena [29].

In the present tumors, KRAS mutation was not confirmed in any of the tumors. However, previous studies have reported the association between KRAS mutation and a high frequency of TB [8, 30]. S100A10 is also considered to contribute to ECM degradation as well as cancer development, invasion, and metastasis via cell surface plasmin generation and RAS cooperation, including KRAS [11, 12, 17]. A few studies reported the relationship between S100A10 and Wnt pathway [31], whereas a recent study suggested that S100A10 is a key regulator of the plasminogen activation system during TGF-β-induced EMT [20].

Actin filaments are associated with cell migration and adhesion, as well as the morphological changes of cells [32]. Previous studies suggested that S100A10 acts as a linker between cytoskeletal actin and cell membrane [18, 19] and that the actin dynamics is strongly regulated by S100A10 [18]. Recently, an actin regulator is involved in PDC progression [33]. In malignant tumors, S100A10 interacts with the ECM proteins that form a structural link with the tumor cell surface [34]. S100A10 is involved in cancer cell detachment by cytoskeletal actin remodeling and in the contacts with convenient ECM for cancer cell invasion by ECM remodeling [11, 17–19, 34].

The ANX A2 belongs to a multigene ANX family of calcium-related and membrane-binding proteins and shows cell-specific expression [13–15, 35]. This protein is involved in diverse cellular functions, such as cell motility/invasion, cell polarity, cell adhesion, and cytoskeletal organization, within the cytoplasm and plasma membranes [13–15, 35]. S100A10 is bound to the tyrosine 23-phosphorylated ANX A2 in the cytoplasm, moves to the cell membrane, and stabilizes [11–17, 35]. More than 90% of ANX A2 is localized at the cell membrane as a subunit of the heterocomplex with S100A10, and the remaining is distributed in the cytoplasm and cell membrane as a monomer [13, 35]. Therefore, membranous S100A10 essentially refers to the S100A10 subunit of the heterocomplex [11–13, 17, 18, 34, 35], which is consistent with the immunohistochemical results of the present tumor. Previously, Graauw et al. indicated that tyrosine 23-phosphorylated, membranous ANX A2 induces cell scattering and branching morphogenesis [36]. Tristante et al. reported a strong membranous immunopositivity for ANX A2 in tumor buds of CRC [37]. These reports may have actually pointed out the features that the S100A10 subunit dominates [12–18].

S100A10 positivity at the luminal surface was also found in the adjacent normal crypt (data not shown). S100A10 expression at the luminal surface has also been observed in the mammary ducts, regardless of whether it is cancerous or noncancerous [23], and is considered to be associated with the establishment and maintenance of polarization of glandular epithelial cells, as one of the functions of S100A10-ANX A2 heterocomplex [23, 38–40]. However, the reason why the protein complex is highly expressed in TB/PDC remains unclear, indicating the collapse of the cell polarity. In poorly differentiated cancer cells, the functions of S100A10-ANX A2 heterocomplex are out of balance and that another function, such as promotion of cell migration/invasion [11, 12, 23, 35, 38, 41], becomes dominant. Hence, future studies will be necessary to confirm the aforementioned research question.

In the present tumors, S100A10 may be poorly related to the stromal cells in TB/PDC because its expression was either absent or very weak. However, recent reports suggested that CAF increase phosphorylated ANX A2 of cancer cells in EMT [42]. The involvement of S100A10 in CAF-induced EMT should be examined in future studies.

This report has several limitations, including patient selection bias, the limited conclusive relationships shown by S100A10 and ANX A2 staining alone, and the intrinsic limitations of a case report.

In conclusion, membranous S100A10 has the potential to be related to TB of CRC during oncogenesis. This may be due to plasminogen activation, actin remodeling, and interaction with an altered ECM [17–19, 34]. It is hypothesized that these functions are more predominant than those of ANX A2 [12–18]. However, further research is required to elucidate differences in the functions of S100A10 and ANX A2.

## Data Availability

The authors declare that all relevant data are included in this published article and are available within the paper.

## Abbreviations

ANX A2: Annexin A2
CA19-9: Cancer antigen 19-9
CAF: Cancer-associated fibroblasts
CEA: Carcinoembryonic antigen
CK: Cytokeratin
CRC: Colorectal cancer
CT: Computed tomography
ECM: Extracellular matrix
EMT: Epithelial-mesenchymal transition
H&E: Hematoxylin and eosin
MMR: Mismatch repair
PDC: Poorly differentiated cluster
PGCC: Polyploid giant cancer cell
TB: Tumor budding
TNM: Tumor, node, and metastasis
